# Within-family influences on dimensional neurobehavioral traits in a high-risk genetic model

**DOI:** 10.1017/S0033291720005279

**Published:** 2022-10

**Authors:** Ania M. Fiksinski, Tracy Heung, Maria Corral, Elemi J. Breetvelt, Gregory Costain, Christian R. Marshall, Rene S. Kahn, Jacob A.S. Vorstman, Anne S. Bassett

**Affiliations:** 1 Department of Psychiatry, Brain Center, University Medical Center Utrecht, Utrecht, The Netherlands; 2 Clinical Genetics Research Program, Centre for Addiction and Mental Health, Toronto, Ontario, Canada; 3 The Dalglish Family 22q Clinic for 22q11.2 Deletion Syndrome, Toronto General Hospital, University Health Network, Toronto, Ontario, Canada; 4 Department of Psychiatry, University of Toronto, Toronto, Ontario, Canada; 5 Division of Clinical and Metabolic Genetics, The Hospital for Sick Children, Toronto, Ontario, Canada; 6 Program in Genetics and Genome Biology, Research Institute, The Hospital for Sick Children, Toronto, Ontario, Canada; 7 Genome Diagnostics, Department of Paediatric Laboratory Medicine, The Hospital for Sick Children, Toronto, Ontario, Canada; 8 Department of Laboratory Medicine and Pathobiology, University of Toronto, Toronto, Ontario, Canada; 9 Department of Psychiatry, Icahn School of Medicine at Mount Sinai, New York, NY, USA; 10 Toronto General Hospital Research Institute and Campbell Family Mental Health Research Institute, Toronto, Ontario, Canada

**Keywords:** 22q11.2 deletion syndrome, genetics, quantitative traits, schizophrenia, shared variance, variable expression

## Abstract

**Background:**

Genotype-first and within-family studies can elucidate factors that contribute to psychiatric illness. Combining these approaches, we investigated the patterns of influence of parental scores, a high-impact variant, and schizophrenia on dimensional neurobehavioral phenotypes implicated in major psychiatric disorders.

**Methods:**

We quantitatively assessed cognitive (FSIQ, VIQ, PIQ), social, and motor functioning in 82 adult individuals with a *de novo* 22q11.2 deletion (22 with schizophrenia), and 148 of their unaffected parents. We calculated within-family correlations and effect sizes of the 22q11.2 deletion and schizophrenia, and used linear regressions to assess contributions to neurobehavioral measures.

**Results:**

Proband-parent intra-class correlations (ICC) were significant for cognitive measures (e.g. FSIQ ICC = 0.549, *p* < 0.0001), but not for social or motor measures. Compared to biparental scores, the 22q11.2 deletion conferred significant impairments for all phenotypes assessed (effect sizes −1.39 to −2.07 s.d.), strongest for PIQ. There were further decrements in those with schizophrenia. Regression models explained up to 37.7% of the variance in IQ and indicated that for proband IQ, parental IQ had larger effects than schizophrenia.

**Conclusions:**

This study, for the first time, disentangles the impact of a high-impact variant from the modifying effects of parental scores and schizophrenia on relevant neurobehavioral phenotypes. The robust proband-parent correlations for cognitive measures, independent of the impact of the 22q11.2 deletion and of schizophrenia, suggest that, for certain phenotypes, shared genetic variation plays a significant role in expression. Molecular genetic and predictor studies are needed to elucidate shared factors and their contribution to psychiatric illness in this and other high-risk groups.

## Introduction

The dimensional study of clinical and behavioral phenotypes can further our understanding of the etiologies of major neuropsychiatric disorders (Nelson, McGorry, Wichers, Wigman, & Hartmann, 2017; Sonuga-Barke, 2014). ‘Genotype-first’ strategies involving specific genetic variants are increasingly recognized as successful routes to this end (Insel, 2010; Lord & Veenstra-VanderWeele, 2016; Moreno-De-Luca, 2016; Moreno-De-Luca et al., 2013). Specifically, selecting a cohort based on a high-impact genetic variant that confers increased risk of psychiatric disorders, such as the 22q11.2 microdeletion for schizophrenia, can provide enhanced etiologic homogeneity and reduced phenotypic ascertainment bias (Bassett et al., 2003; Insel, 2010; Moreno-De-Luca et al., 2013; Stessman, Bernier, & Eichler, 2014; Van, Boot, & Bassett, 2017).

To understand how shared genetic background can shape the expression of neurobehavioral traits in the general population, a standard ‘within-family’ approach involves measuring the averaged outcome of both parents (the biparental mean) (Constantino & Todd, 2005; Devlin, Daniels, & Roeder, 1997). Emerging findings using a within-family approach in individuals with pathogenic variants suggest that parental scores may play a role in explaining the variable phenotypic expressivity of such variants (Malich, Largo, Schinzel, Molinari, & Eiholzer, 2000; Moreno-De-Luca et al., 2015). However, data on most high-impact variants are unavailable as of yet. Focusing on variants conveying risk for later onset phenotypes such as schizophrenia poses particular challenges given the necessity of acquiring data from both adult probands and their parents.

In the current study, we combined genotype-first and within-family approaches to disentangle the relative impact of a *de novo* 22q11.2 deletion, from the modifying effects of parental scores and of schizophrenia on relevant phenotypic measures. The 22q11.2 deletion confers the highest known molecular risk for developing schizophrenia (~25-fold) and is therefore considered a valuable genetic model for studying factors involved in psychotic illness (Bassett et al., 2003; Insel, 2010; McDonald-McGinn et al., 2015; Van et al., 2017). Importantly, individuals with a 22q11.2 deletion, with and without schizophrenia, show impairments in the same heritable traits of cognitive, social and motor functioning (Boot et al., 2015; Fiksinski et al., 2017; Vorstman et al., 2015) as do patients with idiopathic schizophrenia and other at-risk groups (D'Angelo et al., 2019; Mollon & Reichenberg, 2018; Poletti, Gebhardt, Kvande, Ford, & Raballo, 2019). Whether these measurable traits are affected by variation shared with parents is however unknown for the 22q11.2 deletion.

In trios comprising adult probands with a *de novo* 22q11.2 deletion, with and without schizophrenia, and their unaffected parents who do not share the deletion, we used standard assessments to acquire data on relevant neurobehavioral measures. The primary goal was to investigate whether parental variability on these measures would account for a significant proportion of the variability of the same phenotypes in probands. Determining the extent of these within-family (shared) effects using this study design also allowed us to estimate effect sizes relative to expectations based on parental scores, thus providing an assessment of the extent of the deleterious effect of the 22q11.2 deletion, and additional impairment related to schizophrenia expression, for each measure.

## Methods and materials

### Procedure and participants

The families included were recruited through participants in a longitudinal study of adults with 22q11.2 deletion syndrome (22q11DS) (Supplemental Methods) (Bassett et al., 2003). Written informed consent was obtained for all participants and the study was approved by local research ethics boards.

Study participants comprised 230 individuals: 82 adult probands with a *de novo* 22q11.2 deletion [mean age 27.2 (9.0; range 18–55) years; 41 (50.6%) male) and 148 unaffected parents (77 mothers, 71 fathers; *n* = 79 probands)] for within-family analyses (online Supplementary Fig. 1). Mean age of parents was 57.6 (s.d. 9.0; range 39–83) years. We aimed to include as many complete trios as possible, in order to use biparental mean scores of neurobehavioral measures for analyses. However, if data for one parent were unavailable, we included the proband-parent dyad.

Clinical genetic testing confirmed the molecular diagnosis of a typical 22q11.2 deletion for all probands and its absence in all participating parents (details in Supplemental Methods) (McDonald-McGinn et al., 2015).

Standard diagnostic assessment (Supplemental Methods) (Bassett et al., 2003) placed the majority of probands (*n* = 52, 63.4%) in the no psychotic illness subgroup; of these, at assessment *n* = 22 (42.3%) were aged ⩾25 and *n* = 30 (57.7%) 18–25 years. The schizophrenia subgroup comprised 22 (26.8%) probands diagnosed with schizophrenia/schizoaffective disorder, mean (s.d.) age at onset 19.8 (4.2), duration of illness 8.9 (9.4) years; none in an acute psychotic phase of illness at assessment. The remaining *n* = 8 (9.8%) probands had a mood disorder with psychotic features, or history of psychotic symptoms thus were excluded from analyses using the main diagnostic subgroups. No parent had a psychotic illness.

### Assessment instruments

We used the same assessment instruments for all participating probands and parents. These instruments have previously been validated or frequently used in the general population as well as in populations with lower cognitive level, including individuals with 22q11DS (Wechsler, 2011). All assessments of cognitive and motor functioning were administered by trained psychologists and all assessments of social functioning [parent-about-proband and parent-about-parent reports (Supplemental Methods)] were supervised and interpreted by trained psychologists. The number of families for whom both proband and parental data were available differed per instrument, resulting in maximum sample sizes of *n* = 78 families for cognitive (FSIQ, VIQ *n* = 77; PIQ *n* = 78), *n* = 61 families for social, and *n* = 72 for motor, functioning (online Supplementary Fig. 1). Proband-only analyses included three individuals for whom no parental data were available.

To assess the level of cognitive functioning, we used the Wechsler Abbreviated Scale of Intelligence, second edition (WASI-II). (Wechsler, 2011) The WASI-II provides Full Scale IQ (FSIQ), Verbal Comprehension Index [VCI, equivalent to verbal IQ (VIQ)] and Perceptual Reasoning Index [PRI, equivalent to performance IQ (PIQ)] scores, each with the general population mean 100, s.d. 15 (Wechsler, 2011).

To assess the level of social functioning, we used the Social Responsiveness Scale-II (SRS) (Constantino et al., 2003); a 65-item measure assessing overall social impairment. Raw scores (mean 30, s.d. 20) were used for analyses, as these provide optimal differentiation at the lower and higher ends of the scale (Constantino et al., 2003).

To assess motor functioning (dexterity), we used the Purdue Pegboard Test (PPT) (Tiffen, 1968). We used *t*-scores (mean 50, s.d. 10) from the bilateral condition for analyses (details in Supplemental Methods).

### Statistical analyses

Primary analyses examined the association between parental and proband functioning by intraclass correlation analyses (ICC). Where ICC results identified a significant association between parental and proband phenotype we proceeded with a linear regression analysis to investigate the effects of parental scores on the respective proband scores while accounting for possible effects of schizophrenia (Fiksinski et al., 2019; Weinberger et al., 2016), proband sex and proband age.

We also compared scores between probands and parents, and between probands with and without schizophrenia, using related samples *t* tests to investigate the deleterious effects of the 22q11.2 deletion and schizophrenia for all dimensional neurobehavioral domains. We calculated effect sizes, expressed in s.d., of the respective difference scores for all phenotypes in a standardized way to allow for cross-phenotype comparisons.

Further, we investigated whether the effect of parental scores on proband scores was different for probands in the schizophrenia and no psychotic illness subgroups. Within the schizophrenia subgroup we assessed the possible influence of age at onset or illness duration and for the no psychotic illness subgroup we repeated analyses restricting to probands aged ⩾25 years, likeliest to be through age at risk for schizophrenia (Bassett et al., 2003; Van et al., 2017).

To examine potential parental sex effects we repeated analyses separately for mothers and fathers. In trios, we performed ICC analyses to evaluate the association between parental scores within-families. Further, we examined correlations among the three phenotypes, and where appropriate, assessed whether taking this correlation into account made a difference to results by including the correlated phenotype in regression analyses.

All data quality control/preparation and statistical analyses were conducted in R 3.6.2 GUI 1.70 (R Core Team, 2019).

## Results

### Impact of the de novo 22q11.2 deletion, and expression of schizophrenia, on dimensional phenotypes

Compared to their unaffected parents, on all measures assessed there was significantly impaired functioning for adults with a *de novo* 22q11.2 deletion (Table 1, online Supplementary Figs. 2A and 2B). As expected (Fiksinski et al., 2019; Vorstman, Breetvelt, Thode, Chow, & Bassett, 2013), mean scores indicated better functioning in the 22q11.2 deletion no psychotic illness than the schizophrenia subgroup, for all measures (Table 2, online Supplementary Fig. 2C).

**Table 1.** Dimensional neurobehavioral functioning domains in adults with a *de novo* 22q11.2 deletion and their unaffected parents

Domain of functioning	Probands with 22q11DS^a^	Parents^b^	Proband–parent differences^c^					Proband–parent correlation statistics ^c^	
				Paired samples *t* test			Estimated effect size (s.d.) of the 22q11.2 deletion^c^	Intraclass correlation coefficient (ICC)	
	Mean (s.d.)	Mean (s.d.)	Mean (s.d.)	*t*	df	*p*		ICC	*p*
Cognitive^d^									
FSIQ	72.84 (16.61)	104.46 (13.71)	−32.03 (14.56)	−19.30	76	**5.0 × 10^−31^**	−2.14	0.549	**9.9 × 10^−08^**
VIQ	78.31 (16.90)	103.06 (14.12)	−25.19 (15.65)	−14.12	76	**6.0 × 10^−23^**	−1.68	0.496	**1.9 × 10^−06^**
PIQ	71.37 (16.04)	104.72 (12.81)	−33.75 (14.61)	−20.40	77	**8.7 × 10^−33^**	−2.25	0.500	**1.3 × 10^−06^**
Social^e^ (SRS)	67.80 (27.92)	31.43 (20.78)	37.66 (32.08)	9.17	60	**5.2 × 10^−13^**	−1.88	0.161	0.106
Motor^f^ (Purdue)	24.85 (12.39)	41.60 (10.51)	−16.39 (15.05)	−9.24	71	**8.5 × 10^−14^**	−1.64	0.127	0.141

a For the descriptive statistics presented here, all available data for probands were used: FSIQ and VIQ *n* = 81; PIQ *n* = 82; SRS *n* = 66; Purdue *n* = 77 (online Supplementary Fig. 1).

b For the descriptive statistics presented here, all available data for parents were used: FSIQ, VIQ and PIQ *n* = 137; SRS *n* = 116; Purdue *n* = 128 (online Supplementary Fig. 1).

c For the proband-parent difference and correlation analyses, data from families where scores were available for both proband and one or both parents were used (see below and online Supplementary Fig. 1 for details). Overall effect size estimates of the 22q11.2 deletion are based on within-family expectations based on parental scores (regardless of the expression of schizophrenia in the proband). Figure 1 shows relative effects in non-psychotic and schizophrenia proband sub-groups; details of effect sizes are presented in the text.

d For cognitive functioning, FSIQ and VIQ data were available for 77 families: *n* = 58 (75.3%) complete trios (thus biparental mean was used), *n* = 16 (20.8%) proband-mother dyads, and *n* = 3 (3.9%) proband-father dyads. PIQ data were available for 78 families: 59 (75.6%) complete trios (thus biparental mean was used), *n* = 16 (20.5%) proband-mother dyads, and *n* = 3 (3.8%) proband-father dyads.

e For social functioning, SRS data were available for 61 families: *n* = 51 (83.6%) complete trios (thus biparental mean was used), and *n* = 10 (16.4%) proband–father dyads.

f For motor functioning, Purdue data were available for 72 families: *n* = 52 (72.2%) complete trios (thus biparental mean was used), *n* = 15 (20.8%) proband-mother dyads, and *n* = 5 (6.9%) proband–father dyads.

Bold font indicates significance at the *p* < 0.001 level

**Table 2.** Dimensional neurobehavioral functioning domains in adults with a *de novo* 22q11.2 deletion with and without schizophrenia

Domain of functioning	22q11DS No psychotic illness subgroup^a^	22q11DS Schizophrenia subgroup^b^	*t* test
	Mean (s.d.)	Mean (s.d.)	*p*
Cognitive			
FSIQ	75.51 (14.73)	63.86 (16.47)	**0.007**
VIQ	80.94 (14.37)	68.36 (17.13)	**0.005**
PIQ	73.73 (15.50)	64.50 (14.87)	**0.02**
Social (SRS)	59.19 (25.82)	83.88 (24.62)	**0.002**
Motor (Purdue)	27.69 (10.23)	15.70 (11.46)	**0.0002**

a Of the total *n* = 52 probands in the no psychotic illness subgroup, data for FSIQ and VIQ were available in *n* = 51; PIQ *n* = 52; SRS *n* = 43; and Purdue *n* = 49.

b Of the total *n* = 22 probands in the schizophrenia subgroup, data for FSIQ, VIQ, and PIQ were available in *n* = 22; SRS *n* = 16; and Purdue *n* = 21.

Bold font indicates significance at the *p* < 0.05 level.

**Fig. 1.**
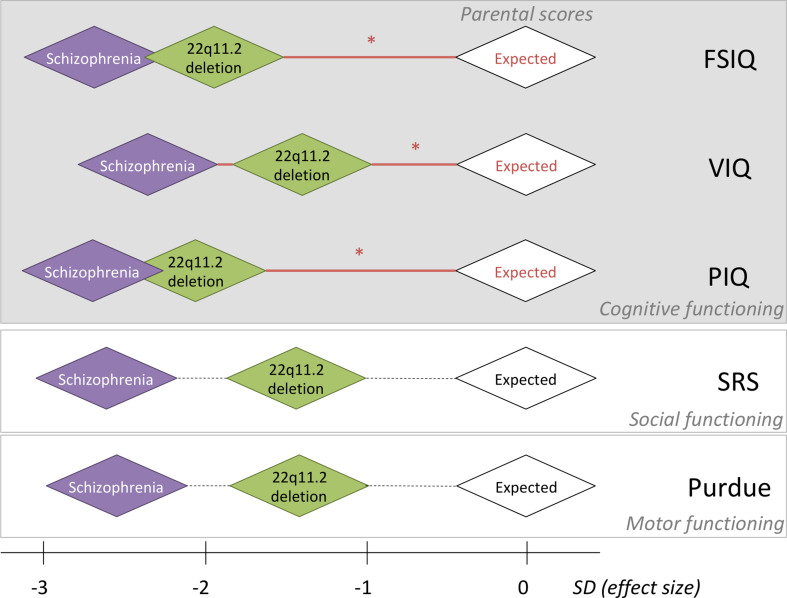
Relative effect sizes of the *de novo* 22q11.2 deletion and schizophrenia in the context of within-family expectations on five-dimensional neurobehavioral traits. For each of five-dimensional neurobehavioral constructs studied, the within-family biparental mean score, indicated by the white diamonds, was set to be standard deviation (s.d.) of 0, representing the average expected score for adult probands with a *de novo* 22q11.2 deletion, based on the respective within-family parental scores. Red horizontal lines with an asterisk indicate significant effects of parental scores on the respective scores assessed in probands; non-significant correlations with parental scores are represented by dashed horizontal lines (details in Tables 1 and 3). Green diamonds are centered at the average estimated effect size (in s.d.) of the *de novo* 22q11.2 deletion on each phenotype, using results for the affected adult probands with the 22q11.2 deletion *and no psychotic illness*; thus, representing the estimated effect size of the 22q11.2 deletion. Purple diamonds are centered at the average estimated effect size (in s.d.) of those with the 22q11.2 deletion and schizophrenia, on each phenotype; thus, indicating the estimated additional effect size of schizophrenia expression. Details about these effect size s.d. are presented in this paper. For simplicity sake, the sizes of all diamond shapes were kept consistent; while pictorially representing general inter-individual variability, they do not represent confidence intervals. Individual within-family results that indicate the preserved relationship for FSIQ between probands with a *de novo* 22q11.2 deletion and their unaffected parents, regardless of cognitive level, are shown for FSIQ in Fig. 2.

**Fig. 2.**
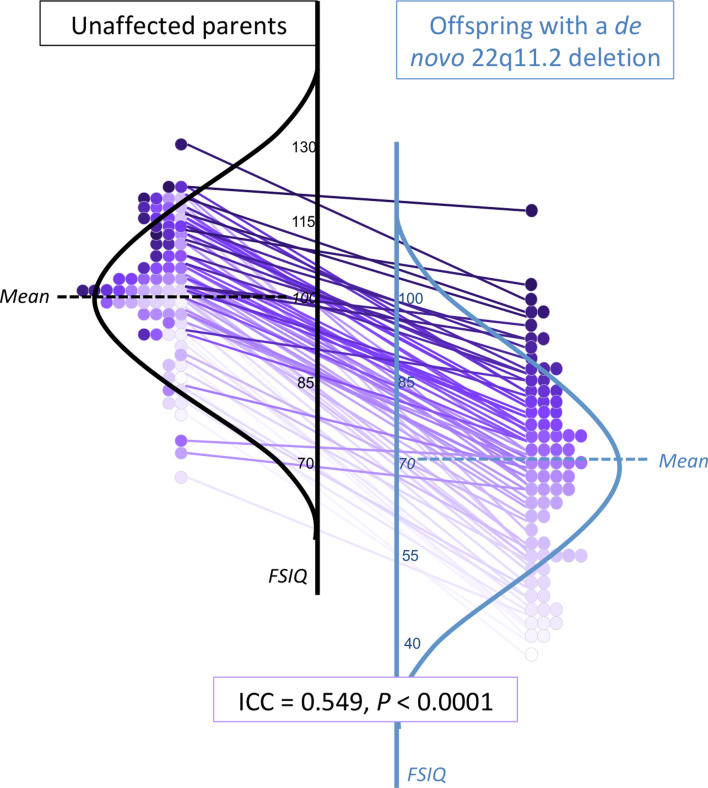
Within-family proband-parent correlation of FSIQ for adults with a *de novo* 22q11.2 deletion. Details of within-family FSIQ data for adult probands with a *de novo* 22q11.2 deletion, each purple-colored circle representing one family (total *n* = 77), ordered by proband FSIQ level (deepest intensity, highest FSIQ), with the same colored circle used for the corresponding biparental FSIQ result and within-family connections indicated by straight lines between each 22q11DS proband-unaffected parent pair. These data are superimposed on schematic depictions of their normalized distributions, for adult probands (blue, right) and their unaffected parents (black, left). While the results for those with a 22q11.2 deletion are on average lower, the individual datapoints and connector lines indicate the overall preserved relationship of FSIQ within families, regardless of proband FSIQ level. Online Supplementary Figs. 2A–2C show the scales shifted so that idealized curves mirror each other, and present schematic representations of FSIQ distributions for the overall sample and for the no psychotic illness and schizophrenia subgroups.

**Table 3.** Relationships between biparental IQ and proband IQ in adults with a 22q11.2 deletion, accounting for schizophrenia status, age and sex in linear regression models^a^

Dependent variable	Variable in model	Coefficient	Standard error (coefficient)	Standardized beta	*t* ratio	*p*
Proband FSIQ^b^	Biparental FSIQ	0.62547	0.111	0.547	5.658	**3.6 × 10^−07^**
	Proband schizophrenia status	−9.651	3.360	−0.280	−2.872	**5.5 × 10^−03^**
	Proband age	−0.078	0.179	−0.043	−0.434	0.666
	Proband sex	2.391	3.102	0.075	0.771	0.443
Proband VIQ^c^	Biparental VIQ	0.6034	0.111	0.541	5.454	**7.9 × 10^−07^**
	Proband schizophrenia status	−12.121	3.377	−0.349	−3.589	**6.3 × 10^−04^**
	Proband age	−0.200	0.186	−0.109	−1.077	0.285
	Proband sex	3.569	3.111	0.111	1.147	0.255
Proband PIQ^d^	Biparental PIQ	0.582	0.129	0.477	4.507	**2.7 × 10^−05^**
	Proband schizophrenia status	−5.672	3.643	−0.167	−1.557	0.124
	Proband age	0.091	0.189	0.050	0.480	0.632
	Proband sex	1.386	3.341	0.044	0.415	0.680

a Total of *n* = 71 families were available for the linear regression analyses for FSIQ and VIQ, i.e. FSIQ and VIQ data for probands (no psychotic illness *n* = 49 (69.0%); schizophrenia *n* = 22 (31.0%)) and their parent(s). Total of *n* = 72 families was available for the linear regression analysis for PIQ, i.e., PIQ data for probands [no psychotic illness *n* = 50 (69.4%)]; schizophrenia *n* = 22 (30.6%) and their parent(s). Mean (s.d.) age of the 72 probands was 26.6 (8.8) years; *n* = 37 (51.4%) were males.

b The overall model for proband FSIQ was significant: R2 = 0.377, *F* = 11.6, *p* *=* 3.4 × 10^−07^.

c The overall model for proband VIQ was significant: R2 = 0.377, *F* = 11.6, *p* *=* 3.5 × 10^−07^.

d The overall model for proband PIQ was significant: R2 = 0.255, *F* = 7.1, *p* *=* 8.1 × 10^−05^.

Bold font indicates significance at the *p* *<* 0.01 level.

Examining the dimensional trait results within these main subgroups allowed us to estimate the relative effect sizes of the *de novo* 22q11.2 deletion and of expression of schizophrenia, anchored by expected results based on parental scores. For those with no psychotic illness, the differences between proband and parental mean scores indicated that the 22q11.2 deletion exerts a deleterious impact of −1.93 s.d. (FSIQ), −1.47 s.d. (VIQ), −2.07 s.d. (PIQ), −1.39 s.d. (SRS), and −1.39 s.d. (Purdue), i.e., substantial for all phenotypes and with the largest effect size for PIQ (Fig. 1).

For the schizophrenia subgroup, estimating from results for the non-psychotic subgroup, expression of schizophrenia added a further decrement, −0.78 s.d. (FSIQ), −0.84 s.d. (VIQ), −0.62 s.d. (PIQ), −1.23 s.d. (SRS), and −1.20 s.d. (Purdue), to the deleterious impact of the 22q11.2 deletion on the phenotypes assessed, i.e. least for PIQ (Fig. 1).

Importantly, the mean (s.d.) parental scores did not differ significantly between the non-psychotic and schizophrenia proband subgroups on any of the phenotypes assessed [FSIQ 104.4 (13.27) *v.* 102.84 (15.68); VIQ 102.05 (12.89) *v.* 103.77 (17.80); PIQ 105.9 (13.08) *v.* 100.55 (12.06); SRS 30.53 (19.75) *v.* 32.56 (22.40); Purdue 42.50 (10.62) *v.* 40.63 (10.88), respectively].

### Phenotypic impact of parental scores within families

Within-family analyses showed highly significant correlations between proband and parental values for each of the three IQ parameters (Table 1, Figs 1 and 2), with effects evident within both the non-psychotic and schizophrenia subgroups (online Supplementary Table 1).

A linear regression model explained 37.7% of the variance in proband FSIQ (*p* *<* 0.001, Table 3), with parental FSIQ and schizophrenia showing independent significant contributions. Results for VIQ were similar, with the model explaining 37.7% of the variance for probands (Table 3). For proband PIQ, the model explained a somewhat lower proportion of the variance (25.5%) and schizophrenia did not reach significance (Table 3). For all three models, parental IQ had the largest effect on proband IQ and proband sex and age were non-significant factors (Table 3).

In contrast to the cognitive traits studied, the within-family proband-parent correlation results for the social and motor phenotypes, although in the expected direction, did not reach significance (Table 1; online Supplementary Table 1).

### Additional analyses

For all phenotypes assessed, within-family parental (i.e. mother-father) values were significantly correlated, most strongly so for FSIQ and VIQ. The ICCs between available maternal and paternal values were: FSIQ 0.640 (*p* *<* 0.001, *n* = 59 pairs), VIQ 0.733 (*p* *<* 0.001), PIQ 0.332 (*p* *=* 0.005), SRS 0.319 (*p* *=* 0.009, 53 pairs), and Purdue 0.368 (*p* *=* 0.003, 53 pairs); online Supplementary Table 1 shows biparental ICC results for non-psychotic and schizophrenia subgroups.

There were no significant parental sex effects on any of the proband phenotypes, nor interaction effects between the predictor variables (probands age, sex, and schizophrenia) in regression models. Limiting the analyses to non-psychotic individuals aged ⩾25 years did not materially impact results. Of the phenotypes assessed in probands, only IQ and Purdue results were significantly correlated with each other (FSIQ *r* = 0.463; VIQ *r* = 0.404; PIQ *r* = 0.484; *p* *<* 0.001 for each). However, incorporating this into analyses did not materially alter results with respect to the ICC and regression models.

## Discussion

In this study, we combined a genotype-first approach and within-family analyses to evaluate the relative effects of parental scores, a high-impact variant, and schizophrenia, on measurable neurobehavioral traits assessed in adult probands with a *de novo* 22q11.2 deletion, with and without schizophrenia. We chose cognitive, social and motor measures as dimensional traits that are relevant to high risk for schizophrenia, including that conveyed by the 22q11.2 deletion, and that are heritable in the general population (Boot et al., 2015; Constantino & Todd, 2005; D'Angelo et al., 2019; Devlin et al., 1997; Fiksinski et al., 2017, 2019; Mollon & Reichenberg, 2018; Poletti et al., 2019; Vorstman et al., 2015). There were several novel findings. Notably, for the cognitive measures, within-family analyses revealed that parental IQ scores maintained a significant and robust correlation to IQ scores in probands that was independent of the effects of the 22q11.2 deletion and of schizophrenia. The modifying effect of parental scores on social and motor measures did not reach significance, suggesting possible differences in genetic architecture for these traits to those for cognitive traits within this etiologically homogeneous high-risk population.

Our study design also allowed us for the first time to estimate the degree to which each variable was affected by the 22q11.2 deletion and by schizophrenia, anchored by expected results based on their unaffected parents' scores. These results indicated that the deleterious impact of the 22q11.2 deletion ranges in effect size from 1.39 to 2.07 s.d. on the phenotypes examined, and that schizophrenia exerts a further negative impact of 0.62–1.23 s.d.. Overall, the latter estimates are consistent with effect sizes reported for cognitive, social and motor measures in idiopathic schizophrenia and schizophrenia high-risk groups (D'Angelo et al., 2019; Mollon & Reichenberg, 2018; Poletti et al., 2019). The pattern of results, taking into account the effects of shared variation based on within-family parental scores, however, revealed further implications for schizophrenia risk in 22q11DS. The findings suggest that the 22q11.2 deletion has the strongest impact on PIQ and the additional impact of schizophrenia expression was weakest for PIQ. These results are consistent with, and extend, previous findings in 22q11DS that PIQ is differentially impaired from a young age, and that VIQ declines over development, but especially so in individuals who go on to develop schizophrenia (Vorstman et al., 2015). Interestingly, there is other evidence from population-based data in support of a genetic risk relationship between schizophrenia and lower PIQ. Hubbard et al. reported a genetic correlation between schizophrenia and PIQ but not VIQ or other cognitive variables (Hubbard et al., 2016) and Lowther et al., reported that pathogenic structural variants (not including 22q11.2 deletions) were especially enriched in individuals with schizophrenia and differentially impaired PIQ (Lowther et al., 2017).

In the context of parental results that were comparable to general population expectations (Constantino et al., 2003; Tiffin & Asher, 1948; Wechsler, 2011), the deleterious effects of the 22q11.2 deletion on cognitive, social and motor functioning identified in the current study are in line with previous findings for the 22q11.2 deletion, in the absence of this parental context (Boot et al., 2015; Fiksinski et al., 2017; Vorstman et al., 2015). Indeed, when accounting for the impact of the 22q11.2 deletion, the within-family proband-parent correlations for IQ approach those observed in the general population for first-degree relatives (Devlin et al., 1997; Plomin & Deary, 2015). The results are broadly consistent with, but extend those of, studies of 22q11DS with parental data that used a proxy for IQ or did not correct for within-family effects (Klaassen et al., 2016; Olszewski, Radoeva, Fremont, Kates, & Antshel, 2014).

In comparing our findings to those for another *de novo* pathogenic variant, the 16p11.2 deletion (Moreno-De-Luca et al., 2015), several observations stand out (online Supplementary Table 2), that are also broadly in line with a study that modeled effect sizes of CNVs on IQ (Huguet et al., 2018). First, although the 22q11.2 deletion and the 16p11.2 deletion both exert deleterious effects across the dimensional phenotypes assessed, the impact of the 22q11.2 deletion appears overall somewhat stronger on cognitive functioning, especially PIQ. Second, the significant within-family effects of parental PIQ on proband PIQ, but not for social functioning assessed using the SRS, differ from findings for the 16p11.2 deletion (Moreno-De-Luca et al., 2015). This may be related to the differential impact of the 22q11.2 deletion on the risk of schizophrenia and of the 16p11.2 deletion on the risk of autism spectrum disorders (Hanson et al., 2015). Methodological differences could also play a role, including diverse cognitive assessment tools from the Simons autism project, younger age at assessment, and higher parental and proband IQs in the 16p11.2 deletion study (Moreno-De-Luca et al., 2015). Nonetheless, the findings collectively suggest that high-impact variants may have differential patterns of relative effect size on the expression of neurobehavioral phenotypes, that may be related to the risk of major neuropsychiatric illness, and to the degree of modification by shared genetic variation.

### Potential implications

The findings have potential implications for clinical care and research. The robust proband-parent correlations for measures of cognitive functioning, regardless of the major effects of the 22q11.2 deletion or of schizophrenia expression, suggest that parental measures, and factors relevant to these measures, could be valuable in developing predictive algorithms for outcomes of individuals with this, and other, high-impact variants. Eventually, such research could be translatable to clinical settings to refine individualized predictions for patients and possibly to suggest ameliorating strategies (Finucane, Challman, Martin, & Ledbetter, 2016; Huguet et al., 2018; Moreno-De-Luca et al., 2015).

The comparability of the results to those for the general population supports the likelihood that common and rare genetic variants, likely to be predominantly inherited, help shape the variable expression of the cognitive phenotype, as they do for the schizophrenia phenotype, in individuals with the 22q11.2 deletion (Davies et al., 2020; Bassett et al., 2017; Cleynen et al., 2020). Indeed, recent proband-only studies from a large international collaboration have demonstrated that polygenic risk scores derived from general population data, for schizophrenia and for cognitive functioning, were significantly associated with expression of these respective phenotypes in individuals with 22q11DS (Cleynen et al., 2020; Davies et al., 2020). Studies that include parental data could further help determine the extent to which such common variant risk, and rare inherited or *de novo* variants, explain the association between parental and proband cognitive functioning, and – importantly – the relationship of these dimensional phenotypes to schizophrenia risk (Bassett et al., 2017; Cleynen et al., 2020; Toulopoulou et al., 2019). The findings could thus inform hypotheses about shared genetic mechanisms that may underlie the expression of schizophrenia and key component dimensional phenotypes, not only in the context of the threshold-lowering 22q11.2 deletion but in other at-risk populations (Insel, 2010; Van et al., 2017).

The fact that the observed within-family correlations for social and motor functioning were not significant may suggest that additional inherited (shared) variants, at least in the context of a 22q11.2 deletion, exert lesser effects on these phenotypes than in the case of IQ, where heritability is high (Plomin & Deary, 2015; Toulopoulou et al., 2019). Additional non-shared factors, environmental and/or perhaps *de novo* genetic variants (Bassett et al., 2017; Cleynen et al., 2020; Vorstman et al., 2017), may play a more prominent role for motor and social traits in 22q11DS.

While not explicitly addressed in our study, the expression of neurobehavioral phenotypes is also likely to be influenced by environmental factors (e.g. SES, neighborhood, and other factors), which may exert an independent impact or interact with genetic factors and mechanisms. For optimal predictive algorithms, future studies that incorporate the potential roles of shared and non-shared genetic and non-genetic factors in individuals from this and other high-risk groups, are warranted (Cleynen et al., 2020; Davies et al., 2020; Van et al., 2016). Collectively, results from such studies will be critical for implementing precision-medicine and promise to eventually contribute to care for individuals with, and at risk for, schizophrenia and other neuropsychiatric disorders (Moreno-De-Luca, 2016; Van et al., 2017).

### Advantages and limitations

Simultaneously assessing multiple dimensional traits in adult probands with a *de novo* 22q11.2 deletion and their unaffected parents, and thereby combining a genotype-first approach with quantitative within-family phenotypic assessments, enabled us to disentangle the impact of this high-impact variant from the modifying effects of corresponding parental scores, and of schizophrenia expression, on the phenotypes assessed. The design and the family-based data used in these analyses are independent of those in previous proband-only studies of 22q11DS (Boot et al., 2015; Cleynen et al., 2020; Davies et al., 2020; Fiksinski et al., 2017, 2019; Vorstman et al., 2015). Standardized estimates of effect sizes not only allow for comparing observed patterns of relative impact across phenotypes within our sample, but also for comparisons with other high-risk populations (D'Angelo et al., 2016; Hippolyte et al., 2016; Moreno-De-Luca et al., 2015; Poletti et al., 2019). Our results set the stage for future studies to formally compare effect sizes of measurable traits between high-impact genetic variants that convey varying levels of risk for neuropsychiatric illnesses. The potential generalizability of our results based on studying probands with a *de novo* 22q11.2 deletion, a minority of whom developed schizophrenia, is supported by the similarity to results reported for other groups at elevated risk for schizophrenia (D'Angelo et al., 2019; Mollon & Reichenberg, 2018; Poletti et al., 2019), and by the similarity of results for their unaffected parents to general population expectations (Constantino et al., 2003; Tiffin & Asher, 1948; Wechsler, 2011).

The main limitation, analogous to that of similar studies (Moreno-De-Luca et al., 2015), relates to sample size. Proband-parent samples are challenging to recruit, especially for adults with a *de novo* high-impact variant and their unaffected parents, thus a minority of the sample comprised proband-parent dyads. Based on the statistical power available to detect the significant effects observed for IQ-parameters, we estimate the minimum detectable effect size of the sample to be ~0.3. Thus proband-parent correlations for social and motor functioning observed to be in the expected direction but non-significant, would be predicted to be small, i.e. less than 0.3. Differences in psychometric properties could also have played a role: the SRS and PP both capture constructs that are narrower and potentially more prone to ‘ceiling-effects’ compared to global cognitive functioning, as assessed with the WASI-II (Constantino et al., 2003; Lord & Veenstra-VanderWeele, 2016; Wechsler, 2011). Nonetheless, a larger number of trios could possibly have allowed the detection of smaller effect sizes.

## Conclusion

The study design, using a unique dataset comprising simultaneous assessments of adult probands with a *de novo* 22q11.2 deletion, with and without schizophrenia, and their unaffected parents, enabled us to dissect the degree to which dimensional neurobehavioral phenotypes implicated in schizophrenia are predicted in probands by the parental scores, by the high-risk genetic variant, and by schizophrenia expression. The results suggest that shared familial variation, likely to include common and rare variants inherited from parents, contributes to shaping the expression of the cognitive phenotype in individuals with a 22q11.2 deletion, with potential implications for the schizophrenia risk conveyed by this high-impact variant. Differing patterns of results for VIQ and PIQ, and for measures of social and motor functioning, suggest differences in genetic architecture, shared and non-shared factors, and effects of schizophrenia expression. Future studies using family-based genome sequencing data will be needed to elucidate the relevant genetic mechanisms involved and which of the shared (inherited) variation identified for cognitive factors overlaps with, and which is separable from the risk of schizophrenia. Improved understanding of the variable phenotypic expression of such a high-impact variant as the 22q11.2 deletion promises to aid delineation of the genetic architecture of schizophrenia in general. Detailed genomic and phenotypic data in the context of molecular high-risk models will complement studies of more heterogeneous samples, helping to converge on mechanisms and pathways to inform precision medicine for all.

## Supplementary material

For supplementary material accompanying this paper visit https://doi.org/10.1017/S0033291720005279. click here to view supplementary material

## Copyright transfer

All authors agree to the submission of this manuscript.
