# Supplementary material for: Within-family influences on dimensional neurobehavioral traits in a high-risk genetic model
Source: Psychol Med. 2021 Jan 14;52(14):3184–92. doi: 10.1017/S0033291720005279 (PMC9693655; doi:10.1017/S0033291720005279)
Supplement: Supplementary file 1 [file S0033291720005279sup001.docx]

# ONLINE SUPPLEMENTAL MATERIAL

***Outline***

- Supplemental Methods.
- Supplemental Table 1. Within-family correlation analyses for the *de novo* 22q11.2 deletion, within the non-psychotic and schizophrenia subgroups.
- Supplemental Table 2. Parent-proband functioning results across five dimensional neurobehavioral phenotypes available from the current study and other studies of the *de novo* 22q11.2 deletion and of the *de novo* 16p11.2 deletion.
- Supplemental Figure 1. Flowchart of participants in the study overall and per dimensional phenotype.
- Supplemental Figures 2A, 2B, and 2C. FSIQ distributions of adult probands with *de novo* 22q11.2 deletions and their unaffected parents exactly as in Figure 2 but without within-family connecting lines and placing idealized curves in mirrored position (2A), and the same idealized schematic representations of FSIQ distributions for probands and parents, but without data points, overall (2B), and for probands with no psychotic illness and schizophrenia (2C).

**Supplemental Methods.**

*Participants and procedure.* All individuals with a molecularly confirmed 22q11.2 deletion and the potential availability of both biological parents were eligible for this study. Despite the potential biases to such family studies where complete trios or even dyads are challenging to recruit, especially for adult patients, the data suggest that our sample is largely representative of the overall population of individuals with the 22q11.2 deletion. Historically, the focus of the clinic (and longitudinal study) through which the participants were recruited was congenital cardiac abnormalities, medical genetics sources, and psychiatric illness. Over time, the center has evolved into a nation-wide specialty clinic for adults with the 22q11.2 deletion, regardless of the phenotypic expression. The participants in this study, therefore, vary in terms of phenotypic expression, including level of overall cognitive functioning (ranging from IQ 44 – 128), as is characteristic of the 22q11DS population.

The *de novo* status of the 22q11.2 deletion was confirmed through genetic testing of both parents for 74 probands; status was deemed probable *de novo* for the remaining 8 probands given that the unavailable co-parent had no features consistent with 22q11DS (1). Of the 79 families, 73 probands had a confirmed de novo status, and the remaining 6 probands were deemed de novo.

Clinical research diagnoses including schizophrenia spectrum disorders were made by experienced clinician-scientists (2), using DSM criteria and information from the Structured Clinical Interview for Diagnostic and Statistical Manual of Mental Disorders, 4^th^ Edition (SCID-IV), direct observation, collateral history from family members, and available lifetime medical records, as previously described (2, 3). For the current study, we derived DSM-5 diagnoses.

*Assessment instruments.* The Wechsler Abbreviated Scale of Intelligence, second edition (WASI-II) is designed to provide a brief and accurate assessment of IQ in individuals ranging in age from 6 to 90 years. It has sound psychometric properties and is used for general population and clinical samples, including those with intellectual disabilities or neurodevelopmental disorders.

For the Social Responsiveness Scale-II (SRS), answers on specific items can range from 1 (not true) to 4 (almost always true). Parents first completed the SRS reporting on their offspring with 22q11DS, and then on the other parent. The raw scores of the SRS can be used to yield standardized scores that indicate social functioning ranging from normal, to mild, moderate or severe impairment.

In the Purdue Pegboard, individuals are presented with 2 cups filled with pins and two vertical rows of 25 holes. Individuals are instructed to place as many pins as possible in 30 seconds down the row on the side of their dominant hand, then their non-dominant hand, and then both hands simultaneously.

*R-packages.* All statistical analyses were performed using R 3.6.2 GUI 1.70, and we made use of the additional R-packages “plyr”, “psych”, “ICC”, “irr”, “pwr”, “lme4”, and “lm.beta”.

**Supplemental Table 1. Within-family correlation analyses in the non-psychotic and schizophrenia subgroups of adult probands with *de novo* 22q11.2 deletions.**

|  | Intra-class correlation (ICC) statistics | | | | | | | |
| --- | --- | --- | --- | --- | --- | --- | --- | --- |
|  | **Non-psychotic 22q11.2 deletion proband subgroup ^a^** | | | | **Schizophrenia 22q11.2 deletion proband subgroup ^b^** | | | |
|  | **Proband-parent** | | **Biparental (mother-father)** | | **Proband-parent** | | **Biparental (mother-father)** | |
|  | **ICC** | p | **ICC** | ***p*** | **ICC** | ***p*** | **ICC** | ***p*** |
| FSIQ | 0.526 | **4.4e-05** | 0.583 | **5.0e-05** | 0.668 | **2.5e-04** | 0.782 | **1.7e-04** |
| VIQ | 0.456 | **4.4e-04** | 0.655 | **3.1e-06** | 0.684 | **1.6e-04** | 0.860 | **9.8e-06** |
| PIQ | 0.475 | **2.1e-04** | 0.335 | **0.019** | 0.506 | **6.8e-04** | 0.251 | 0.174 |
|  |  |  |  |  |  |  |  |  |
| SRS | 0.124 | 0.223 | 0.420 | **0.005** | 0.112 | 0.340 | -0.130 | 0.664 |
|  |  |  |  |  |  |  |  |  |
| Purdue | 0.043 | 0.387 | 0.394 | **0.009** | 0.332 | 0.071 | 0.246 | 0.209 |

^a^ Of the total n = 52 probands in the non-psychotic subgroup, proband-parent data were available for ICC analyses for n = 49 families for FSIQ and VIQ; n = 50 families for PIQ; n = 39 families for SRS; and n = 46 families for Purdue. Biparental (mother-father) ICC analyses included n = 38 pairs for FSIQ, VIQ, and PIQ; n = 35 pairs for SRS and Purdue.

^b^ Of the total 22 probands in the schizophrenia subgroup, proband-parent data were available for ICC analyses for n = 22 families for FSIQ, VIQ, and PIQ; n = 15 families for SRS; and n = 20 families for Purdue. Biparental (mother-father) ICC analyses included n = 15 pairs for FSIQ, VIQ, and PIQ; n = 12 pairs for SRS and Purdue.

Bold font indicates significance at the *p* < 0.05 level.

**Supplemental Table 2. Parent-proband functioning results across five dimensional neurobehavioral phenotypes – Current and previous studies of *de novo* 22q11.2 deletion and *de novo* 16p11.2 deletion.**

|  | **FSIQ** | | | | **VIQ** | | | | **PIQ** | | | |
| --- | --- | --- | --- | --- | --- | --- | --- | --- | --- | --- | --- | --- |
|  | Parental scores | Proband scores | Deletion impact (SD) | Proband-parent correlation | Parental scores | Proband scores | Deletion impact (SD) | Proband-parent correlation | Parental scores | Proband scores | Deletion impact (SD) | Proband-parent correlation |
|  | Mean (SD) | Mean (SD) |  |  | Mean (SD) | Mean (SD) |  |  | Mean (SD) | Mean (SD) |  |  |
| *Current study (22q11.2 deletion)^a^* | 104 (14) | 73 (17) | -2.1 | ICC = 0.55 | 103 (14) | 78 (17) | -1.7 | ICC = 0.50 | 105 (13) | 71 (16) | -2.3 | ICC = 0.50 |
|  |  |  |  | ***p* = 9.9e-08** |  |  |  | ***p* = 1.9e-06** |  |  |  | ***p* = 1.3e-06** |
| *2014 22q11.2 deletion study ^b^* | 101 (12) | 72 (14) | -1.9 ^d^ | * | - | - | - | * | - | - | - | * |
|  |  |  |  | ***p* < 0.01** |  |  |  | ***p* < 0.01** |  |  |  | ***p* < 0.01** |
| *2015 16p11.2 deletion study ^c^* | 112 (10) | 86 (15) | -1.7 | ICC = 0.42 | 108 (9) | 83 (17) | -1.6 | ICC = 0.53 | 114 (10) | 88 (17) | -1.7 | ICC = 0.20 |
|  |  |  |  | ***p* = 0.03** |  |  |  | ***p* = 0.003** |  |  |  | *p* = 0.21 |

|  | **Social (SRS)** | | | | **Motor (Purdue)** | | | |
| --- | --- | --- | --- | --- | --- | --- | --- | --- |
|  | Parental scores | Proband scores | Deletion impact (SD) | Proband-parent correlation | Parental scores | Proband scores | Deletion impact (SD) | Proband-parent correlation |
|  | Mean (SD) | Mean (SD) |  |  | Mean (SD) | Mean (SD) |  |  |
| *Current study (22q11.2 deletion) ^a^* | 31 (21) ^e^ | 68 (28) | -1.9 | ICC = 0.15 | 42 (11) | 25 (12) | -1.6 | ICC = 0.13 |
|  |  |  |  | *p* = 0.11 |  |  |  | *p* = 0.14 |
| *2014 22q11.2 deletion study ^b^* | - | - | - | - | - | - | - | - |
| *2015 16p11.2 deletion study ^c^* | 30 (18) | 75 (33) | -2.2 | ICC = 0.52 | 42 (9) | 30 (16) | -1.3 | ICC = 0.21 |
|  |  |  |  | ***p* = 0.009** |  |  |  | *p* = 0.22 |

^a^ Current study; Maximum n = 82 probands with a *de novo* 22q11.2 deletion, mean age = 27.2 (9.0) y; see details in text, Table 1 footnote, and eFigure 1 for details of sample sizes for within-family correlation analyses for each phenotype assessed; ICC results include probands with psychotic illness

^b^ Olszewski et al., 2014(4); Maximum n = 69 probands with a *de novo* 22q11.2 deletion, mean age = 18.0 (2.2) y; ICC results include probands with psychotic illness/symptoms. * No biparental data were reported in the Olszewski et al., 2014 study; correlation coefficient R values reported for proband-mother data were: FSIQ R = 0.599, VIQ R = 0.546, PIQ R = 0.437. Note that R is not corrected for within-family effects, hence is not directly comparable to ICC values.

^c^ Moreno-De-Luca et al., 2015(5); Maximum n = 54 probands with a *de novo* 16p11.2 deletion, reported age >2 y; ICC results include probands with autism spectrum disorder

^d^ Estimated from data reported in Olszewski et al., 2014

^e^ Standardized SRS T-scores: Of the biparental SRS results, 90.2% were within “normal limits” based on population norms; of the proband SRS results, 48.4% were within “normal limits”, 46.9% were in the “mild-to-moderate”, and 4.7% were in the “severe” range.

Bold font indicates statistical significance at the *p* < 0.05 level.

NB. General population means for FSIQ, VIQ, and PIQ = 100 (SD = 15); for SRS mean = 30 (SD = 20); and for Purdue mean = 50 (SD = 10).

**Supplemental Figure 1. Flowchart of participants in the study overall and per dimensional phenotype.**


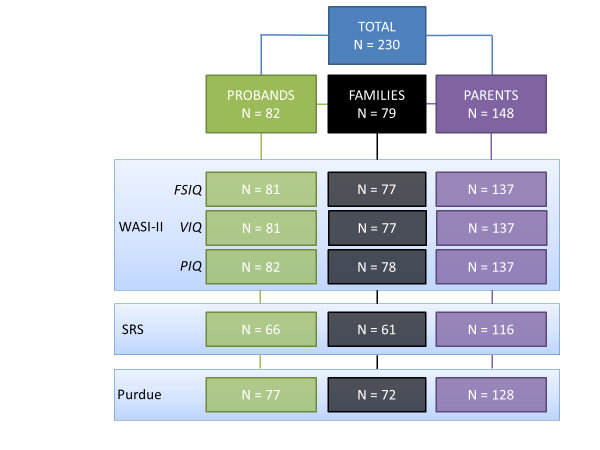


**Supplemental Figure 2A. Detailed within-family FSIQ distributions for adult probands with *de novo* 22q11.2 deletions and their unaffected parents data-point color intensity corresponding to proband FSIQ, with idealized distribution curves in mirrored position (see Figure 2 for further details and within-family connector lines)**


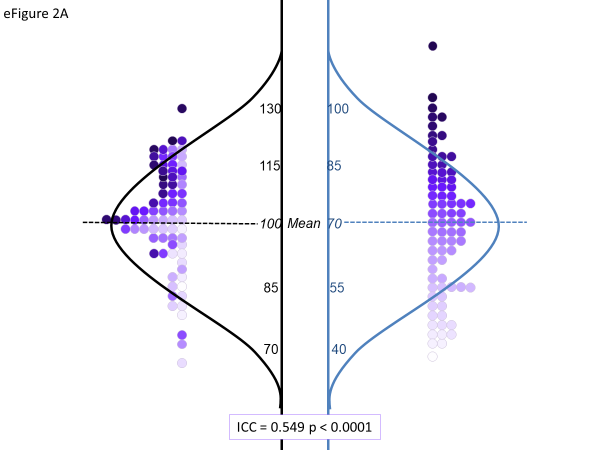


**Supplemental Figures 2B and 2C. Idealized schematic representations of FSIQ distributions of probands with *de novo* 22q11.2 deletions and unaffected parents, overall (2B), and probands in no psychotic illness and schizophrenia subgroups (2C).**


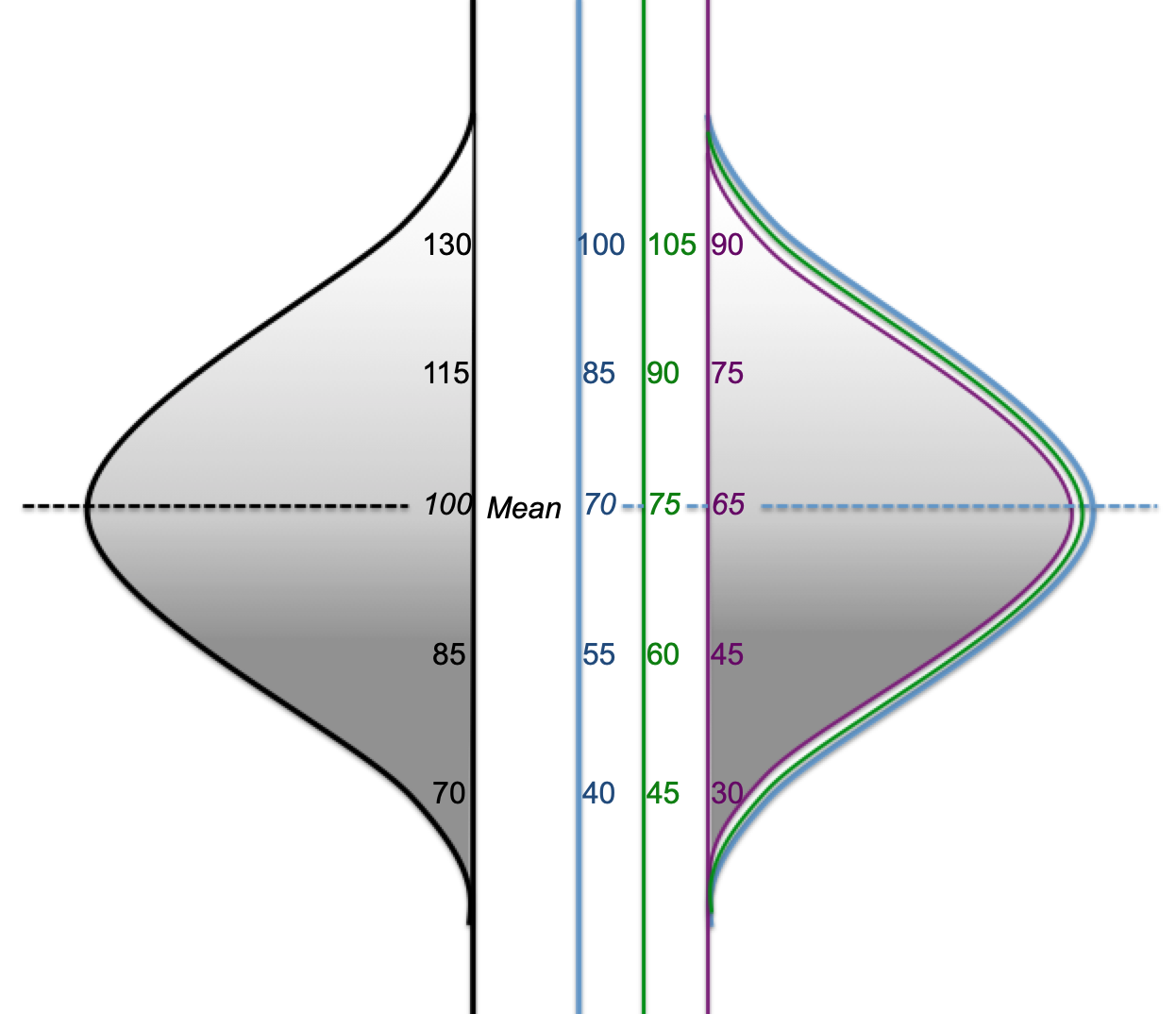

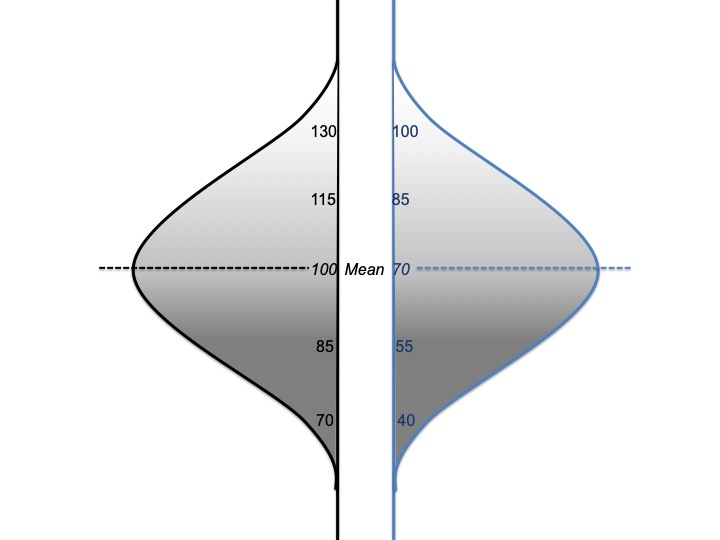


**2C.** Idealized schematic depiction of distribution of FSIQ in adult probands with a *de novo* 22q11.2 deletion (right), with no psychotic illness, and with schizophrenia, and their unaffected parents (left).

*As for the total group of individuals with a 22q11.2 deletion, the significant correlation between parental and proband IQ is preserved, both in those with schizophrenia (mean IQ ≈ 65, SD 15), and in those with no psychotic illness (mean IQ* *≈ 75, SD 15).* *n IQ ≈ 75, SD 15).*

IQ

distribution

Individuals with a *de novo* 22q11.2 deletion

No psychotic illness

With schizophrenia

Unaffected parents of individuals with 22q11DS

**2B.** Idealized schematic depiction of distribution of FSIQ in adult probands with a *de novo* 22q11.2 deletion (right) and their unaffected parents (left).

*Despite the shifted IQ-scale in the individuals with a 22q11.2 deletion (average ~30 IQ points), the distribution is not shifted randomly but closely corresponds to the distribution of IQs of their respective parents.*

IQ

distribution

Unaffected parents of individuals with 22q11DS

Individuals with a *de novo* 22q11.2 deletion

REFERENCES

1. Van L, Heung T, Graffi J, Ng E, Malecki S, Van Mil S, et al. (2019): All-cause mortality and survival in adults with 22q11.2 deletion syndrome. *Genet Med*. 21:2328-2335.

2. Bassett AS, Chow EW, AbdelMalik P, Gheorghiu M, Husted J, Weksberg R (2003): The schizophrenia phenotype in 22q11 deletion syndrome. *Am J Psychiatry*. 160:1580-1586.

3. Fiksinski AM, Breetvelt EJ, Lee YJ, Boot E, Butcher N, Palmer L, et al. (2018): Neurocognition and adaptive functioning in a genetic high risk model of schizophrenia. *Psychol Med*. 49:1047-1054.

4. Olszewski AK, Radoeva PD, Fremont W, Kates WR, Antshel KM (2014): Is child intelligence associated with parent and sibling intelligence in individuals with developmental disorders? An investigation in youth with 22q11.2 deletion (velo-cardio-facial) syndrome. *Res Dev Disabil*. 35:3582-3590.

5. Moreno-De-Luca A, Evans DW, Boomer KB, Hanson E, Bernier R, Goin-Kochel RP, et al. (2015): The role of parental cognitive, behavioral, and motor profiles in clinical variability in individuals with chromosome 16p11.2 deletions. *JAMA Psychiatry*. 72:119-126.
